# Rapid EST isolation from chromosome 1R of rye

**DOI:** 10.1186/1471-2229-8-28

**Published:** 2008-03-18

**Authors:** Ruo-Nan Zhou, Rui Shi, Shu-Mei Jiang, Wei-Bo Yin, Huang-Huang Wang, Yu-Hong Chen, Jun Hu, Richard RC Wang, Xiang-Qi Zhang, Zan-Min Hu

**Affiliations:** 1 Institute of Genetics and Developmental Biology, Chinese Academy of Sciences, Beijing 100101, P. R. China; 2 South China Sea Institute of Oceanology, Chinese Academy of Sciences, Guangzhou 510301, P. R. China; 3 USDA-ARS, FRRL, Utah State University, Logan, UT 84322-6300, USA; 4 Graduate University of Chinese Academy of Sciences, Beijing 100049, P. R. China; 5 Forest Biotechnology Group, N.C. State University, Campus Box 7247, Raleigh, NC 27695-7247, USA

## Abstract

**Background:**

To obtain important expressed sequence tags (ESTs) located on specific chromosomes is currently difficult. Construction of single-chromosome EST library could be an efficient strategy to isolate important ESTs located on specific chromosomes. In this research we developed a method to rapidly isolate ESTs from chromosome 1R of rye by combining the techniques of chromosome microdissection with hybrid specific amplification (HSA).

**Results:**

Chromosome 1R was isolated by a glass needle and digested with proteinase K (PK). The DNA of chromosome 1R was amplified by two rounds of PCR using a degenerated oligonucleotide 6-MW sequence with a *Sau*3AI digestion site as the primer. The PCR product was digested with *Sau*3AI and linked with adaptor HSA1, then hybridized with the *Sau*3AI digested cDNA with adaptor HSA2 of rye leaves with and without salicylic acid (SA) treatment, respectively. The hybridized DNA fragments were recovered by the HSA method and cloned into pMD18-T vector. The cloned inserts were released by PCR using the partial sequences in HSA1 and HSA2 as the primers and then sequenced. Of the 94 ESTs obtained and analyzed, 6 were known sequences located on rye chromosome 1R or on homologous group 1 chromosomes of wheat; all of them were highly homologous with ESTs of wheat, barley and/or other plants in *Gramineae*, some of which were induced by abiotic or biotic stresses. Isolated in this research were 22 ESTs with unknown functions, probably representing some new genes on rye chromosome 1R.

**Conclusion:**

We developed a new method to rapidly clone chromosome-specific ESTs from chromosome 1R of rye. The information reported here should be useful for cloning and investigating the new genes found on chromosome 1R.

## Background

EST analysis has opened up exciting prospects for gene discovery in all organisms, irrespective of their genome size [1-7]. Large-scale mapping of EST unique genes can provide valuable insights into the organization of genomes and chromosomes [8]. EST distribution in relation to chromosome landmarks (short and long arms, euchromatin, heterochromatin, centromeres, and telomeres) and recombination is important in comparative analysis of chromosome structure and evolution, gene isolation, and targeted genome sequencing for large genome species such as wheat [8-10]. At present, most ESTs are from cDNA libraries, but EST identification and localization are laborious and time consuming, especially for polyploid plants because of the large genome size and serious interference of homologous sequences. Construction of single-chromosome or chromosome-region EST library could be an efficient strategy to isolate important ESTs located on specific chromosomes and/or specific chromosomal regions.

There are several reports on isolation of ESTs directly from specific chromosomes and/or specific chromosomal regions, most of which were on EST isolation from human chromosomes or chromosome segments by using microdissected chromosome DNA as probes to screen ESTs from a cDNA library [11,12]. ESTs of microdissected chromosomes had been isolated successfully by using microdissection-mediated cDNA capture [13,14].

Plant chromosome microdissection and microcloning have been studied for more than 10 years. Many chromosome-specific DNA libraries from different plant species, such as wheat [15,16], oat [17], barley [18] and beet [19], have been constructed using this strategy. Our group has constructed several plant chromosome- and chromosome region-specific DNA libraries and has isolated ESTs that are disease-related genes [20-25].

As an important genetic resource of major cereal crop species, rye (*Secale cereale *L., 2n = 14, genome R) has good adaptability to extreme climatic and soil conditions. Rye is also known to have the lowest requirements for chemical treatments like fertilizers or pesticides, which makes it an ecologically and economically desirable crop for specific regions, for example, in northern Europe [26]. Chromosome 1R, which has been shown to carry genes for resistance to powdery mildew [27], stem rust [28], leaf rust [29], yellow rust [30], and greenbug [31], especially attracted scientists' attention.

To date, a high-resolution linkage map of rye has not been established owing to its large genome (approximately 9000 Mb) [32], high content of repetitive sequences and the insufficiency of molecular markers. For the same reasons, constructing the EST map of the whole rye genome is inefficient and time consuming.

It is well known that rye chromosome 1R contains a large number of resistance genes. SA is a critical signal for the activation of both local and systemic acquired resistance (SAR) and can induce the expression of some resistance genes (R genes) [33]. Recent evidence suggests that SA also regulates cell death, possibly via a positive feedback loop that involves reactive oxygen species [34,35].

HSA technique is based on the suppressive PCR principle, which selects and amplifies the common sequences of two complex DNA samples. The use of oligonucleotide adaptors that form strong clamps ensures the specificity of the method, such that only fragments with two different adaptors will be amplified, whereas fragments with one type of adaptor will not be selected for amplification due to the suppressive effect on PCR of the adaptors [36]. In this research, we developed a new method to rapidly isolate ESTs from rye leaves, with and without SA induction, by using the microdissection of chromosome 1R combined with HSA. The method developed in this research to clone ESTs of specific chromosome has not been reported before. It would be a useful method to investigate genes on specific chromosomes. The obtained ESTs reported here should be useful to further clone the new genes on chromosome 1R.

## Results

### Procedures to isolate expressed sequences of a specific chromosome

**Figure 1:**
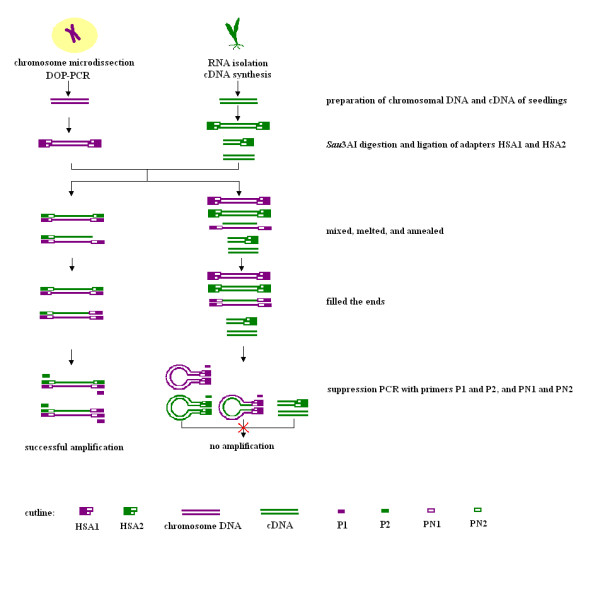
**Procedure to isolate chromosome specific expressed sequences**. The new method that combines chromosome microdissection and HSA to isolate expressed sequences from a specific chromosome is diagrammed.

The procedures of this method are shown in Figure 1. The strategy of this method is the combination of chromosome microdissection method and HSA technique [36] to yield the homologous sequences between microdissected DNA and cDNA. As shown in Figure 1, after degenerated oligonuleotide-primed PCR (DOP-PCR) of microdissected chromosomes, amplified microdissect DNA and cDNA are digested by *Sau*3AI and linked with two kinds of adaptors, respectively. The adaptors and primers described above are designed from suppression subtractive hybridization (SSH) [37], with a change of the blunt ends into annealing ends of *Sau*3AI. The two samples were separately denatured and annealed for 10 h, then mixed and annealed together for another 10 h. Three kinds of hybrid chains were generated: DNA-DNA hybrid chains, DNA-cDNA hybrid chains, and cDNA-cDNA hybrid chains. Finally, a two-step PCR amplification is performed to select the hybridized fraction of the samples. Only the DNA-cDNA hybrid chains, which came from different samples with different adaptors, could be exponentially amplified. Because of the palindrome structure of the adaptors, the DNA-DNA and cDNA-cDNA chains formed the panhandle structure during the amplification. Thus, they could not be further amplified.

### Microdissection of chromosome 1R, its DNA amplification and characterization

**Figure 2:**
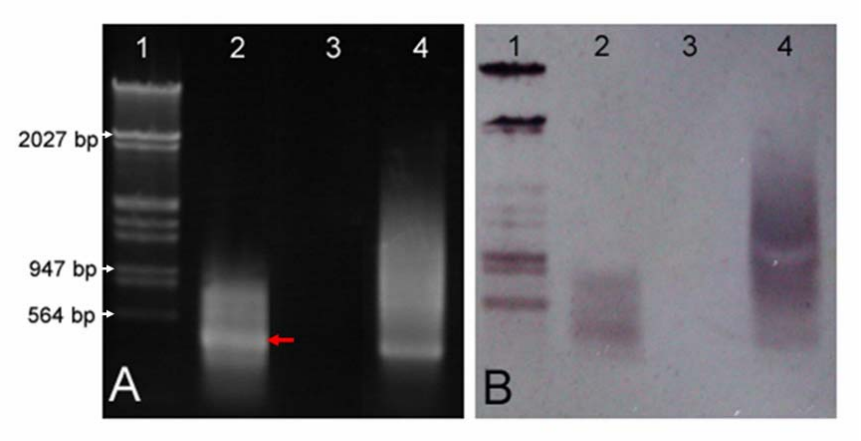
**Products of the second round PCR using DNA of microdissected chromosome 1R of rye**. Products of the second round PCR using microdissected chromosome 1R DNA as the template and degenerated oligonucleotide sequence as the primer (2A), and Southern hybridization of the PCR products using DIG-labeled rye genomic DNA as probe (2B). Lane 1:λDNA digested with *Hin*dIII/*Eco*RI; Lane 2: the second round PCR products of chromosome 1R; Lane 3: negative control without any template DNA; Lane 4: positive control (DOP-PCR products using rye genomic DNA as the template).

Five 1R chromosomes were successfully microdissected and amplified with two rounds of DOP-PCR. The size of the second round PCR products ranged from 0.15 to 1.2 kb, with predominant fragments in the range of 0.25–0.8 kb (Figure 2A, lane 2). The positive control, using 10 pg of genomic rye DNA as template, had a brighter and wider band ranging in size from 0.6 to1.0 kb (Figure 2A, lane 4). No product was obtained from the negative control (Figure 2A, lane 3), which did not contain any added template DNA so that was used to monitor any possible contamination.

In order to verify the origin of PCR products, the amplified products were hybridized with DIG-labeled rye genomic DNA. Hybridization signals were observed only in the positive control and the amplified products from chromosome 1R (Figure 2B), indicating that the microdissected chromosomes came indeed from the rye genome. Furthermore, the intense band for amplified chromosome 1R product in an agarose gel (Figure 2A, lane 2, red arrowed) was recovered, purified and sequenced. The fragment of 300 bp in size was obtained. When compared with the data in GenBank, it was identical to the dispersed repeat sequence R173-1 (GenBank accession number X64100) of *Secale cereale*, which was previously located on chromosome 1RS [38]. This confirmed that the amplified DNA product was truly from microdissected chromosome 1R.

### Hybridization between chromosome 1R DNA and cDNA of rye and suppression amplification

**Figure 3:**
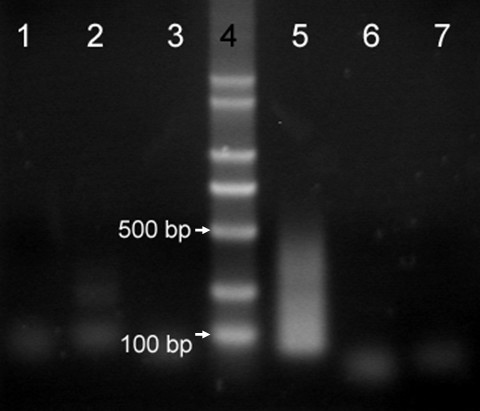
**Electrophoretic patterns of two rounds of suppression PCR**. Electrophoretic patterns of two rounds of suppression PCR with the hybridized DNA/cDNA, between chromosome 1R DNA and cDNA of rye without SA induction, as template. (Lanes 1, 2 and 3) the primary amplification with double primers P1 and P2, single primer PN1 and PN2, respectively; (Lane 4) molecular weight marker-DL2000; (Lane 5) the secondary amplification with double primers PN1 and PN2; (Lanes 6 and 7) amplification with single primer PN1 and PN2, respectively.

The chromosome 1R DNA generated by DOP-PCR was linked with adaptor HSA1 and hybridized with HSA2-linked cDNA of rye plants with and without SA treatment, respectively. After hybridization, the hybridized fragments between chromosome 1R DNA and cDNA of rye were amplified by two rounds of suppression PCR using single primer P1 or P2, double primers P1 + P2, single primer PN1 or PN2, and double primers PN1 + PN2. P1 and PN1 were corresponding to HSA1, and P2 and PN2 were corresponding to HSA2. The relationship between or among those primers was shown in Figure 1. There were no evident PCR products when single primer P1 or P2 or double primers P1 + P2 were used in the first round of PCR. In the second round PCR, there were neither evident PCR products when single primer PN1 or PN2 was used, whereas PCR products ranging from approximately 80 bp to 500 bp were obtained when double primers PN1 + PN2 were used (Figure 3). These results indicated that hybridization and suppression were successful in the experiment.

### Generation of ESTs of chromosome 1R and analysis of sequenced ESTs

**Figure 4:**
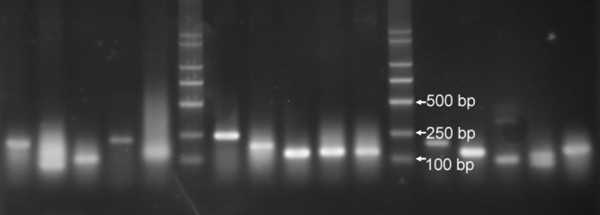
Sampled PCR products released from recombinant clones by using PN1 and PN2 as the primers.

PCR products amplified with double primers PN1 and PN2 were cloned into pMD18-T vector (TaKaRa, Dalian, China). There were about 100 recombinant clones in each plate. A total of 113 recombinant clones were randomly selected from the 1R-chromosome EST libraries of rye leaves with and without SA induction. Of these, 40 clones were from SA-induced leaves. The inserts were released by PCR with PN1 and PN2 primers and sequenced (Figure 4). The 113 sequenced inserts, excluding the primer sequences, ranged from 52 to 411 bp.

Of the 113 sequenced inserts, 4 were contaminated sequences (bacterial DNA sequence), 15 redundant ones, and 94 unique ones. These were named, registered, and analyzed with a Blast search in the GenBank/EMBL database. Results are listed in Additional files 1 and 2. Chromosome 1R DNA possessed primer PN1, whereas rye cDNAs possessed primer PN2. Those sequences possessed both primers PN1 and PN2 (the hybrid products of rye cDNA and 1R DNA, thus, expressed sequences of the chromosome 1R) were successfully isolated.

Of the 94 unique inserts, 60 of them were expressed without SA treatment (Additional file 1) and 34 of them were SA-induced (Additional file 2). These sequences were divided into two classes: 72 known sequences, which are homologous with existing EST sequences in the GenBank database (identities > 80%), and 22 unknown sequences, which had no hits in the GenBank/EMBL database. Of the known sequences, all are homologous with ESTs or genes from rye, wheat and barley; 6 have been located to specific chromosomes (Table 1) – 3 on chromosome 1R of rye [38-40], one on all 7 chromosomes of rye [41], and 2 on homologous group 1 of wheat [42,43]. This unequivocally demonstrated that the ESTs of chromosome 1R could be isolated by our newly developed method.

**Table 1:** Six 1R chromosome-originated ESTs.

Clone No.	dbEST_Id	GenBank_Accn	Length (bp)	Definition	Score	E value	Identities	Sequence property
N8	43782944	EH412046	291	*Secale cereale *thioredoxin-like protein (1R)	490	2.00E-135	271/279 97%	polyA
N17	43782957	EH412059	152	*Triticum turgidum *A genome HMW glutenin A gene locus, sequence (1A)	54	2.00E-04	51/59 86%	
N21	43782961	EH412063	195	EST from *Secale cereale *(1R)	101	1.00E-18	66/71 92%	polyA
N38	43782977	EH412079	89	*Secale cereale *OPH20 RAPD marker sequence (chromosomes 1R-7R)	109	1.00E-21	63/65 96%	
SA2	43783003	EH412105	208	*Secale cereale *omega secalin gene (1RS)	98	9.00E-47	142/154 92%	
SA7	43783019	EH412121	219	*Triticum turgidum *HMW-glutenin locus (1B)	141	1.00E-30	183/219 83%	

Among the recombinant clones, the redundancy is 11% (15/113). The most frequently sequenced inserts were rye clone F17 hypervariable DNA sequences, genes encoding barley beta-ketoacyl-ACP synthase [44], and barley phosphate transporter HvPT4.

**Figure 5:**
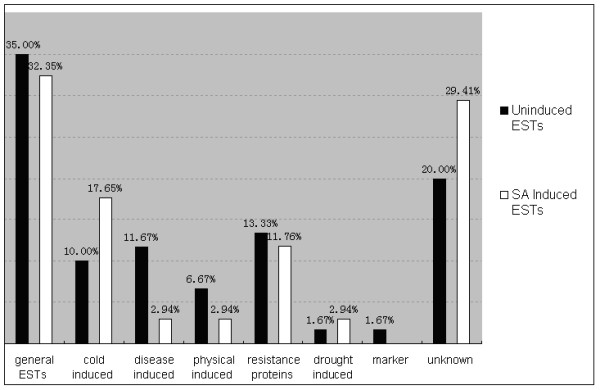
Functional categories of non-SA-induced and SA-induced ESTs.

Of the 60 sequences obtained from leaves of rye plants without SA induction, 45% were genes related to biotic and abiotic stresses, 35% were not related to biotic and abiotic stresses, and 20% were unknown. Compared with the functional categories of ESTs from rye leaves without SA induction, the ESTs related to temperature induction and the unknown ESTs were increased by 7.65% and 9.41%, respectively, in SA-treated plants; whereas general ESTs, the disease-induced ESTs and resistance protein (RP) decreased by 2.65%, 8.73% and 1.57%, respectively (Figure 5).

### Characterization of recombinant clones by dot blot hybridization

**Figure 6:**
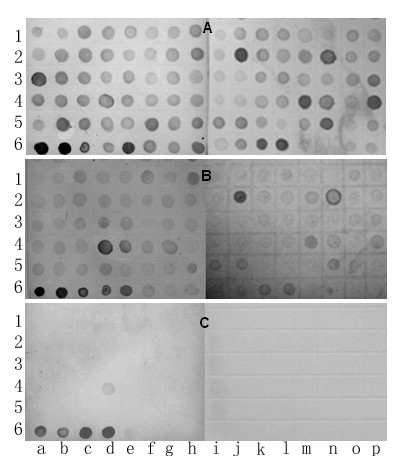
**Characterization of a part of selected recombinant clones by dot blot hybridization**. Characterization of partially selected recombinant clones by dot blot hybridization with DIG-labeled chromosome 1R secondary DOP-PCR product (A), cDNA of rye seedlings at three-leaf-stage induced by salicylic acid (B) and rye genomic DNA (C). 6a, 6b, 6c, 6d and 6e are PCR products of microdissected 1R chromosome, cDNA, rye genomic DNA, wheat genomic DNA, and HSA products, respectively.

The recombinant plasmid DNAs of randomly selected recombinant clones were hybridized with DIG-labeled chromosome 1R secondary DOP-PCR product, cDNA of rye seedlings at three-leaf-stage with SA induction, and rye genomic DNA. Nearly all (99%) recombinant plasmid DNAs could be hybridized with chromosome 1R DOP-PCR product (Figure 6A), indicating that the inserts in recombinant plasmid DNAs are homologous with chromosome 1R DNA. The hybridization signals were weaker when the recombinant plasmid DNAs were hybridized with cDNA of rye seedlings, although some clones had stronger signals (Figure 6B). Not coincidental, these are those clones having stronger signals in hybridization with chromosome 1R DOP-PCR product. Whereas, there were only very weak and/or no hybridization signals when the recombinant clones were hybridized with rye genomic DNA (Figure 6C). These results indicate that all the recombinant clones are present in the rye genome in single and/or low copy, a characteristic for expressed genes.

## Discussion

EST analysis is an efficient way to clone important new genes controlling biotic and abiotic stress resistance and other desirable traits in plants [45,46]. Millions of ESTs from plants have been registered in the GenBank database. At present, most ESTs are from cDNA libraries of different tissues at different developmental stages. However, EST identification and mapping is laborious and time consuming, especially for polyploid plants because of the large genome size and serious interference of homologous sequences. Many important genes have been located on specific chromosomes and/or specific regions of chromosomes by traditional genetics and modern molecular techniques [47,48]. Therefore, cloning ESTs from specific chromosomes or specific regions of chromosomes could be an efficient method to obtain the desirable genes.

In this research we developed a new method to clone the ESTs from a specific chromosome of plants. Some of the amplified DNAs should contain PolyA tail and some did not, but all were expressed genes derived from the microdissected chromosome 1R of rye. Our results prove that this strategy is correct and working. All 94 isolated unique fragments have the primer sequences from two different adaptors, 53 of the 94 fragments are ESTs with PolyA tail and 41 are without. Six sequences had been located on chromosome 1R of rye and/or on homologous group 1 chromosomes of wheat. This clearly demonstrated that the ESTs of chromosome 1R could be isolated by our newly developed method.

Several methods to isolate chromosome-expressed sequences (CES) or ESTs had been previously reported. First, in the probe screening method, CES were obtained by dot hybridization on membranes using DNA fragments derived from chromosome- specific library as probes to screen cDNA library or using cDNA fragments as the probe to screen chromosome-specific library. By using this method, CES had been successfully isolated from humans and animals [12,49-52]. Although the reliability is high, this method is time consuming because of the numerous cloning operations and hybridized clone selections. Second, in the hybrid selection method [11], CES were isolated using cDNAs to hybridize genomic DNA pools that were generated by microdissection of the human chromosome 12qHSR region and immobilized on a nylon membrane, followed by PCR amplification of hybridized fragments. The chromosome DNA was fixed on the membrane to avoid the numerous cloning operations, and the selection sensitivity was improved. But due to the complexity of cDNA, more false positives would occur and many low-abundance cDNA would be lost. Wei *et al*. [51] discovered that only 1/3 of clones were single/low-copy fragments by using this method. Third, in the microdissection-mediated cDNA capture method, the CES could be obtained using cDNA with adaptors to hybridize the chromosomes *in situ *on coverslips, followed by microdissection of hybridized chromosomes and/or specific chromosome region and subsequent amplification using the adaptors-linked cDNA as the primers [13,14,53]. Avoiding the amplification of chromosome DNA, this method is rapid and ingenious but the false positive rate is high. Actually, both of the last two methods are suitable for the CES enrichment. In our newly developed method that is based on chromosome microdissection, DNA-cDNA hybridization in liquid and suppressive PCR, many ESTs or CES can be simultaneously isolated. Compared with the first three above-mentioned methods, our method is more convenient, economical, efficient, and easy to control. In our method, ESTs or CES can be obtained without DNA (or cDNA) library construction and dot (or *in situ*) hybridization.

In previous works, chromosome-specific DNAs were mainly amplified by linker-adaptor PCR (LA-PCR) and DOP-PCR. By LA-PCR, amplified chromosome-specific DNAs were usually longer, but the coverage ratio was lower, than those obtained by DOP-PCR [17]. We used DOP-PCR to amplify chromosome 1R DNA to obtain expressed sequences ranging from 52 to 411 bp in length. Most of these sequences provided enough information to be compared with ESTs in the GenBank database, although they are shorter than those obtained by the probe screening method [51,52].

**Figure 7:**
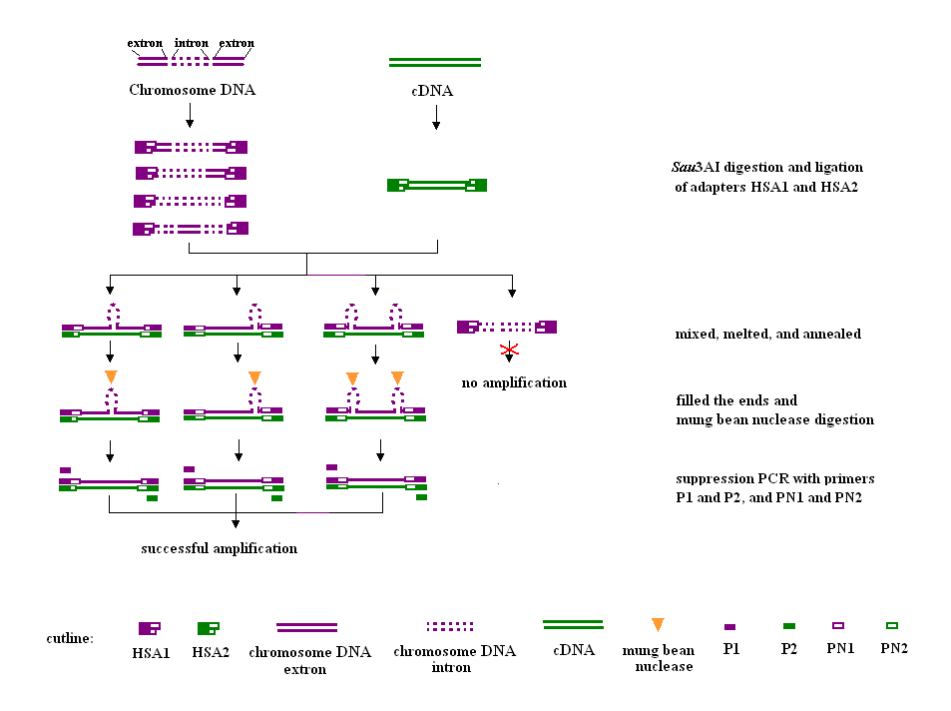
**Procedures to remove the introns of the DNA/cDNA hybrid product**. In the procedures, the mismatch sensitive mung bean nuclease was used to avoid the influence of the introns in HSA.

DNA-cDNA hybridization always suffers from a common imperfection: mismatched strands will frequently appear due to the introns of genes. This disadvantage led to observations that the hybridized sequences were not all necessarily the expressed sequences [36]. In this study, we used the single-strand-specific DNA and RNA endonuclease mung bean nuclease to cleave the partially mismatched strands. Figure 7 shows the entire procedure of clearing up the introns of the hybrid products by the mismatch-sensitive mung bean nuclease. Mung bean nuclease has high specificity for single-stranded DNA and RNA; it is the enzyme of choice for most applications requiring a single-strand-specific nuclease [54]. Theoretically, the introns will not hybridize with cDNAs and will form loops, and the loops will finally be digested by the mung bean nuclease, and the influence of the introns could be avoided. In this research, none of the isolated ESTs contained introns. It was shown that the mung bean nuclease digested the introns efficiently. In all previous research on isolating CES or ESTs, this endonuclease was not used to solve the problem.

During chromosome microdissection, contamination that originates from any DNA of cytoplasm and other species is always a serious problem [55]. Amplification of contaminating DNA will greatly influence the quality of chromosome-specific DNA libraries. However, the sequencing results here did not show any serious contamination originating from alien DNA. The reason might be that only the hybridized DNA-cDNA sequences could be detected and amplified during the procedure of the HSA. The contaminant DNAs could not be amplified because they could not be hybridized with chromosome 1R DNA and/or cDNA of rye leaves. Our results suggested that the HSA technique in our newly developed method could suppress the amplification of contaminant DNAs.

SA is known to be a signal molecule in acquired resistance to pathogens in several species [56,57]. It can be used to induce the synthesis of certain pathogenesis-related (PR) proteins and to enhance resistance to pathogens [58,59]. Janda *et al*. [60] discovered that the addition of 0.5 mM SA to the hydroponic growth solution of young maize plants under normal growth conditions provided protection against subsequent low temperature (2°C) stress. In our study, SA induction significantly increased the ESTs related to temperature induction and the unknown ESTs, whereas decreased general ESTs, the disease-induced ESTs and resistance protein (RP). The SA treatment time may be the main reason for the reduced numbers of ESTs associated with disease resistance because different genes may need different durations of SA treatment to achieve significant expression [61]. In this research, we only use a single treatment duration (24 h) for the SA treatment. Presumably, the treatment for 24 h may be unsuitable for the expression of some disease resistance genes. Moreover, the fact that the unknown ESTs increased after SA induction should be intriguing. Some of these unknown ESTs may be new genes associated with disease resistance. They were not classified into the resistance genes because their functions were unknown. These unknown ESTs need to be investigated.

Finding new genes and detecting their functions are the goals of most genetic research. In fact, the unknown sequences are the most interesting part of this research. In this study, we got 22 unknown sequences (with no hits in the GenBank database) and 72 known sequences, which shared high-degrees of homology with existing EST sequences in the GenBank database. The former provide opportunities to clone new genes from chromosome 1R of rye. Although the method we described here has the disadvantage of having short inserts, the combination of microdissection and microcloning with a recently developed technique, polymerase cloning (Ploning), could be a potential way to solve this problem. Zhang *et al*. [62] reported a Ploning method, which is a sequencing strategy that eliminates culturing of microorganisms by using real-time isothermal amplification to form plones from the DNA of a single cell. They reported that the single-cell *E. coli *library had 96.2% coverage of the genome and with inserts up to 12 kb. By using phi29 polymerase and the N6 primer, the plones led to less amplification bias and larger insert fragments [62].

## Conclusion

In conclusion, an efficient way to clone ESTs from a specific chromosome of a plant was established in this research. It would be a useful method for genomic and functional genomics research of plants, especially for the polyploidy species. Ninty-four ESTs from the chromosome 1R of rye were cloned, sequenced and analyzed. These sequences should be useful in gene expression studies of chromosome 1R of rye plants at the seedling stage either with or without SA induction. Based on our results, it should also be possible to clone the new genes on chromosome 1R and elucidate their functions in future studies.

## Methods

### Plant materials

The plant material used in this research was *Secale cereale *L. var. King II (2n = 14), which was kindly provided by Prof. Terry Miller, John Innes Center, UK.

### Genomic DNA extraction, mRNA isolation and cDNA synthesis

Rye was planted and their leaves at three-leaf stage were sprayed with 1 μg/L SA or water (control). Total RNA was isolated from young leaves of plants 24 h after the treatment, using the Trizol Kit (Invitrogen, Carlsbad). mRNA purification was done with the protocol of PolyATtract mRNA Isolation System III (Promega, Madison). Double stranded cDNA was generated with the cDNA Synthesis Kit (TaKaRa, Dalian). Rye genomic DNA was extracted from young leaves by using the CTAB method [63].

### Chromosome 1R isolation and its DNA amplification

Chromosome 1R isolation and its DNA amplification were performed mainly according to the procedures described earlier [25]. Briefly, the rye seeds were immersed in water (25°C) for 5–8 h, then germinated on moist filter paper in a petri dish at 25°C in dark. After the seeds sprouted, they were cultured at 4°C for 24 h, then at 25°C in dark until the roots grew to 0.5–1 cm in length. The seeds with roots were treated in ice water (0°C) for 24 h to increase metaphase cells. Then root tips were harvested and fixed in 3:1 ethanol: acetic acid for 5 min, transferred immediately into 70% ethanol and stored at -20°C. Before being squashed, the root tips were digested with an enzyme mixture of 2% cellulase (Yakukt, Tokyo) and 2% pectolyase (Dingguo, Beijing) in 75 mM KCl, 7.5 mM EDTA at 37°C for 1 h, then rinsed in double distilled water and stored at 4°C for 15–20 min. After the root tips were squashed in a drop of 1% Carbol Fuchsin solution, they were immediately used for microdissection. The chromosome 1R, identified by its satellite on the short arm [64], was microdissected by using the glass needle fixed on the arm of a LeitZ micro-operation instrument on an inverted phase-contrast microscope (Olympus 1 M, Japan). 5 dissected 1R chromosomes were collected into a 0.2 ml Eppendorf tube and digested with a 10 μl drop of PK buffer [0.5 mg/ml PK (Roche, Indianapolis) in 1°C Taq Polymerase buffer (Promega, Madison)]. Subsequently, chromosome 1R DNAs were amplified by PCR using degenerated oligonucleotide (DOP) sequence as the primer, which was designed according to the degenerated primer 6 MW [65] with a little modification by changing the restriction site *Xho*I into *Sau*3AI. The designed DOP primer sequence is 5'- CCGACTGATCNNNNNNATGTGG -3'. Two rounds of PCR were performed. The first round of PCR was carried out in the same tube by adding 4 μl of 10°C Taq Polymerase buffer (Promega, Madison), 3 μl of 25 mM MgCl_2 _(Promega, Madison), 2.5 U Taq DNA Polymerase (Promega, Madison), 1 μl of 10 mM dNTPs (MBI, Lithuania), 0.7 μl of DOP primer (50 ng/μl) and varying amount of double distilled water to make up the 50 μl final volume. After an initial denaturation (94°C, 10 min), PCR was performed with 5 low-stringency cycles of 94°C for 1 min, 30°C for 1.5 min, and 72°C for 3 min, then followed by 25 high-stringency cycles of 94°C for 1 min, 55°C for 1 min, and 72°C for 1.5 min and a final extension at 72°C for 10 min. The second round of PCR was done under the same conditions described above except that only a 5 μl product from the first round of PCR was used as the template and without the 5 low-stringency cycles. In all the procedures, strict positive and negative control experiments were carried out using the same conditions but the templates. In the positive control experiment, 10 pg of rye genomic DNA were used as the template; whereas in the negative control no DNA template was used.

### Characterization of amplified chromosome 1R DNA

In order to confirm the origin of the amplified microdissection DNA, rye genomic DNA was used as the probe. Rye leaf DNA was isolated by the CTAB method [66]. Probe preparation, southern blotting, and hybridization and signal detection were conducted according to the protocol of DIG DNA Labeling and Detection Kit (Roche, Indianapolis). Furthermore, the obvious band in the amplified microdissection DNA separated in an agarose gel was recovered and purified with the DNA Purification and Recycling Kit (Dingguo, Beijing) and then sequenced.

### ESTs generation from 1R chromosome

#### Adaptors

Adaptors HSA1 and HSA2 were designed according to the sequences for SSH [37] with a slight modification by changing the blunt end into asymmetric end (GATC) recognizable by *Sau*3AI.

Adaptor HSA1

A1L, 5'-GTAATACGACTCACTATAGGGCTCGAGCGGCCGCCCGGGCAGGT-3'

A1S, 3'-CCCGTCCACTAG-5'

Adaptor HSA2

A2L, 5'-TGTAGCGTGAAGACGACAGAAAGGGCGTGGTGCGGAGGGCGGT-3'

A2S, 3'-CCTCCCGCCACTAG-5'

#### Primers

We designed two pairs of primers according to the sequences of HSA1 and HSA2. P1 and PN1 are corresponding to HSA1, and P2 and PN2 are corresponding to HSA 2.

P1, 5'-GTAATACGACTCACTATAGGGC-3'

P2, 5'-TGTAGCGTGAAGACGACAGAA-3'

PN1 (nest primer of P1), 5'-TCGAGCGGCCGCCCGGGCAGGT-3'

PN2 (nest primer of P2), 5'-AGGGCGTGGTGCGGAGGGCGGT-3'

### Hybridization of chromosome 1R DNA and cDNA of rye

The DNA of 1R chromosome (1 μg) and cDNA of rye (1 μg) were digested by 10 U *Sau*3AI (TaKaRa, Dalian) in 25 μl volume at 37°C overnight, respectively, followed by *Sau*3AI inactivation at 70°C for 15 min. Digested 1R DNA and cDNA of rye were linked with HSA1 and HSA2 in 20 μl volume, respectively, by mixing 2.5 μl of *Sau*3AI digested 1R DNA or cDNA of rye, 2 μl (10 μM) of adaptor HSA1or HSA2, 2 μl 10°C T4 ligase buffer (TaKaRa, Dalian), and 1 μl (3.5 U/μl) T4 ligase (TaKaRa, Dalian) and kept at 16°C overnight. After ligation, samples were heated at 70°C for 15 min to inactivate the ligase. The samples with HSA1 and HSA2 were precipitated and resuspended in 2 μl hybridization buffer (50 mM HEPES, pH 8.3; 0.5 M NaCl; 0.02 mM EDTA, pH 8.0; 10% PEG 8000, W/V), then denatured at 98°C for 10 min and incubated at 68°C for 10 h. Subsequently, two samples were mixed together as soon as possible and hybridized at 68°C for 10 h. Hybridized DNA-cDNA duplexes of 1R DNA and cDNA of rye were filled in the ends by using Taq DNA polymerase (TaKaRa, Dalian, 1 U/μl of hybridized DNA) at 72°C for 20 min.

### Hybridized DNA treatment with mismatch-sensitive nucleases

In order to digest the possible mismatched fragments in DNA-cDNA duplexes, aliquots of 100 ng of hybridized DNA-cDNA were treated with 0.1 U of mung bean nuclease (TaKaRa, Dalian) at 37°C for 15 min. The treated sample was purified by phenol/chloroform extraction, and precipitated according to the previously described method [67].

### PCR amplification of Hybridized DNA-cDNA

After digestion by mung bean nuclease, the hybrid product was dissolved in 100 μl of double distilled water and 1 μl of this was used as the template for PCR amplification following adding 2 μl of primers P1 and P2 (5 μM, each 1 μl), 2.5 μl of 10x Taq reaction buffer (Tianwei, Beijing), 1 μl of Taq DNA polymerase (2.5 U/μl, Tianwei, Beijing), and 18.5 μl of double distilled water in a final 25 μl volume. After an initial denaturation at 95°C for 2 min, the PCR was performed with 30 cycles of 30 s at 94°C, 30 s at 68°C, 90 s at 72°C, followed by a 10-min final extension at 72°C. The PCR product was diluted by double distilled water in a ratio of 1:10. Subsequently, the nested PCR was carried out in 25 μl volume with 1 μl of diluted PCR product, 2 μl of PN1 and PN2 (5 μM, each 1 μl), 2.5 μl of 10x Taq reaction buffer (Tianwei, Beijing), 1 μl of Taq DNA Polymerase (2.5 U/μl, Tianwei, Beijing), under the same conditions mentioned above, except that a 25-cycles was used.

### Cloning and analysis of ESTs from chromosome 1R

The nested PCR products were phenol-chloroform extracted, ethanol precipitated and then dissolved into 40 μl of double distilled water. 4 μl of purified second round PCR product was ligated into pMD18-T vector (TakaRa, Dalian). A total of 100 μl transformed *E. coli *DH5α competent cells were transferred onto three fresh ampicillin-containing plates of an 11-cm diameter. The plasmids in randomly selected recombinants were extracted by alkaline lysis with SDS: mini-preparation method [68] and the inserts were released by PCR using PN1 and PN2 primers. The released ESTs were sequenced by Invitrogen (Beijing).

To characterize the recombinants, the plasmid DNAs (each 0.1 ng) were doted onto Hybond N^+ ^filters (NEN Research, Boston, Mass.) that were then hybridized with DIG-labeled secondary DOP-PCR product of chromosome 1R, cDNAs of rye seedlings at three-leaf-stage with SA induction, or rye genomic DNA. The dot blot hybridization was performed according to the manufacturer's instruction of DIG DNA Labeling and Detection Kit (Roche, Indianapolis Cat. No 1093657).

### Data analysis

The DNA sequences and their deduced amino acid sequences obtained in this research were compared with those in the GenBank database using the Blast search programs [69].

## Abbreviations

CES – chromosome-expressed sequences; DIG – digoxigenin; DOP-PCR – degenerated oligonucleotide-primed PCR; ESTs – expressed sequence tags; HSA – hybrid specific amplification; PK – proteinase K; Ploning – polymerase cloning; PN1 – nest primer of P1; PN2 – nest primer of P2; R genes – resistance genes; RP – resistance protein; SA – salicylic acid; SAR – systemic acquired resistance; SSH – suppression subtractive hybridisation.

## Authors' contributions

RNZ carried out the chromosome microdissection, hybridization and statistical analysis, participated in the sequence alignment and drafted the manuscript. RS participated in the design of the study. SMJ and YHC performed the statistical analysis. WBY carried out a part of hybridization work. HHW participated in a part of chromosome microdissection. JH participated in the sequence alignment. XQZ helped in the drafting of manuscript. RRCW obtained the rye seed and thoroughly revised the manuscript. ZMH participated in the design of the study and drafted the manuscript.

## Supplementary Material

Additional file 1 Sequence analysis of ESTs on 1R chromosome of rye plant without SA induction. The data provided the information of sequenced and analyzed ESTs on 1R chromosome of rye plant without SA induction. Click here for file

Additional file 2 Sequence analysis of ESTs on 1R chromosome of rye plant with SA induction. The data provided the information of sequenced and analyzed ESTs on 1R chromosome of rye plant with SA induction. Click here for file

## References

[B1] Adams MD, Kelley JM, Goceyne JD, Dubnick M, Polymeropoulos MH, Xiao H, Merril CR, Wu A, Olde B, Moreno RF, Kerlavage AR, McCombie WR, Venter JC (1991). Complementary DNA sequencing: espressed sequence tags and human genome project. Science.

[B2] Covitz PA, Smith LS, Long SR (1998). Expressed sequence tags from a root-hair-enriched *Medicago truncatula *cDNA library. Plant Physiol.

[B3] Ewing RM, Kahla AB, Poirot O, Lopez F, Audic S, Claverie JM (1999). Large-scale statistical analyses of rice ESTs reveal correlated patterns of gene expression. Genome Res.

[B4] Fernandes J, Brendel V, Gai X, Lal S, Chandler VL, Elumalai RP, Galbraith DW, Pierson EA, Walbot V (2002). Comparison of RNA expression profile based on maize expressed sequence tag frequency analysis and micro-array hybridization. Plant Physiol.

[B5] Hillier L, Lennon G, Becker M, Bonaldo MF, Chiapelli B, Chisoe S, Dietrich N, DuBuque T, Favello A, Gish W (1996). Generation and analysis of 280,000 human expressed sequence tags. Genome Res.

[B6] Shoemaker R, Keim P, Vodkin L, Retzel E, Clifton SW, Waterston R, Smoller D, Coryell V, Khanna A, Erpelding J, Gai X, Brendel V, Raph-Schmidt C, Shoop EG, Vielweber CJ, Schmatz M, Pape D, Bowers Y, Theising B, Martin J, Dante M, Wylie T, Granger C (2002). A compilation of soybean ESTs: generation and analysis. Genome.

[B7] Van der Hoeven R, Ronning C, Giovannoni J, Martin G, Tanksley S (2002). Deductions about the number, organization, and evolution of genes in the tomato genome based on analysis of large expressed sequence tag collection and selective genomic sequencing. Plant Cell.

[B8] Qi LL, Echalier B, Chao S, Lazo GR, Butler GE, Anderson OD, Akhunov ED, Dvoøák JA, Linkiewicz M, Ratnasiri A, Dubcovsky J, Bermudez-Kandianis CE, Greene RA, Kantety R, La Rota CM, Munkvold JD, Sorrells SF, Sorrells ME, Dilbirligi M, Sidhu D, Erayman M, Randhawa HS, Sandhu D, Bondareva SN, Gill KS, Mahmoud AA, Ma XF, Miftahudin, Gustafson JP, Conley EJ, Nduati V, Gonzalez-Hernandez JL, Anderson JA, Peng JH, Lapitan NLV, Hossain KG, Kalavacharla V, Kianian SF, Pathan MS, Zhang DS, Nguyen HT, Choi DW, Fenton RD, Close TJ, McGuire PE, Qualset CO, Gill BS (2004). A Chromosome Bin Map of 16,000 Expressed Sequence Tag Loci and Distribution of Genes Among the Three Genomes of Polyploid Wheat. Genetics.

[B9] Hossain KG, Kalavacharla V, Lazo GR, Hegstad J, Wentz MJ, Simons K, Gehlhar S, Rust JL, Syamala RR, Obeori K, Suresh B, Karunadharma P, Chao S, Anderson OD, Qi LL, Echalier B, Gill BS, Linkiewicz AM, Ratnasiri A, Dubcovsky J, Akhunov ED, Dvorák J, Miftahudin, Ross K, Gustafson JP, Sidhu D, Dilbirligi M, Gill KS, Peng JH, Lapitan NLV, Greene RA, Bermudez-Kandianis CE, Sorrells ME, Feril O, Pathan MS, Nguyen HT, Gonzalez-Hernandez JL, Conley EJ, Anderson JA, Fenton D, Close TJ, McGuire PE, Qualset CO, Kianian SF (2004). A chromosome bin map of 2148 expressed sequence tag loci of wheat homoeologous group 7. Genetics.

[B10] Linkiewicz AM, Qi LL, Gill BS, Echalier B, Chao S, Lazo GR, Hummel DD, Anderson OD, Akhunov ED, Dvorák J, Pathan MS, Nguyen HT, Peng JH, Lapitan NLV, Miftahudin, Gustafson JP, La Rota CM, Sorrells ME, Hossain KG, Kalavacharla V, Kianian SF, Sandhu D, Bondareva SN, Gill KS, Conley EJ, Anderson JA, Fenton RD, Close TJ, McGuire PE, Qualset CO, Dubcovsky J (2004). A 2500-locus bin map of wheat homoeologous group 5 provides insights on gene distribution and colinearity with rice. Genetics.

[B11] Su YA, Trent JM, Guan XY, Meltzer PS (1994). Direct isolation of genes encoded within a homogeneously staining region by chromosome microdissection. Proc Natl Acad Sci USA.

[B12] Zhang M, Yu L, Hu PR, Bi AD, Xia JH, Deng HX, Zhao SY (1997). Isolation of the expression fragments with the probe pool of human chromosome 14q24.3 generated by microdissection. Acta Biologiae experimetalis sinica.

[B13] Gracia E, Ray ME, Polymeropoulos MH, Dehejia A, Meltzer PS, Trent JM (1997). Isolation of chromosome-specific ESTs by microdissection-mediated cDNA capture. Genome Res.

[B14] Kim H, Kim C, Rhyu IJ, Kim JH, Lew DH, Chun YH, Park SH (2001). Isolation of novel human fetal brain cDNAs mapped to human chromosome bands, 1q25 and 8q24.1. Mol Cells.

[B15] Albani D, Côté MJ, Armstrong KC, Chen QF, Segal A, Robert LS (1993). PCR amplification of microdissected wheat chromosome arms in a 'single tube' reaction. Plant J.

[B16] Liu B, Segal G, Vega JM, Feldman M, Abbo S (1997). Isolation and characterization of chromosome-specific DNA sequences from a chromosome arm genomic library of common wheat. Plant J.

[B17] Chen Q, Armstrong K (1995). Characterization of a library from a single microdissected oat (*Avena sativa *L.) chromosome. Genome.

[B18] Busch W, Martin R, Herrmann RG, Hohmann U (1995). Repeated DNA sequences isolated by microdissection I. Karyotyping of barley (*Hordeum vulgare *L.). Genome.

[B19] Jung C, Claussen U, Horsthemke B, Fis-cher F, Herrmann RG (1992). A DNA library from an individual *Beta patellaris *chromosome conferring nematode resistance obtained by microdissection of meiotic metaphase chromosome. Plant Mol Biol.

[B20] Dang BY, Hu ZM, Zhou YH, Cui LH, Wang LL, Zhang TH, Li LC, Chen ZH (1998). Isolation of single-chromosome DNA library from *Lilium regale wilson*. Chin Sci Bull.

[B21] Jiang SM, Hu J, Yin WB, Chen YH, Wang RRC, Hu ZM (2005). Cloning of resistance gene analogs located on the alien chromosome in an addition line of wheat-Thinopyrum intermedium. Theor Appl Genet.

[B22] Jiang SM, Zhang L, Hu J, Shi R, Zhou G, Chen YH, Yin WB, Wang RRC, Hu ZM (2004). Screening and analysis of differentially expressed genes from an alien addition line of wheat Thynopyrum intermedium induced by barley yellow dwarf virus infection. Genome.

[B23] Zhang FY, Yin WB, Shi R, Hu YK, Yan YM, Chen YH, Zhou YH, Hu J, Wang RRC, Hu ZM (2005). Construction and characterization of chromosome 1B specific DNA library of wheat. Cana J Plant Sci.

[B24] Zhou YH, Dang BY, Wang H, Hu ZM, Wang LL, Chen ZH (2001). Microdissection of a single chromosome and construction of t-he microclone library from soybean. Euphytica.

[B25] Zhou YH, Hu ZM, Dang BY, Wang H, Deng XD, Chen ZH (1999). Microdissection and microloning of rye (*Secale cereale *L.) chromosome 1R. Chromosoma.

[B26] Kubaláková M, Valárik M, Bartoš J, Vrána J, Cíhalíková J, Molnár-Láng M, Doleže J (2003). Analysis and sorting of rye (*Secale cereale *L.) chromosomes using flow cytometry. Genome.

[B27] van Kints TMC (1986). Mildew of wheat. UK Cereal Pathogen Virulence Survey. Annual Report.

[B28] Mago R, Spielmeyer W, Lawrence GJ, Ellis JG, Pryor AJ (2004). Resistance genes for rye stem rust (*SrR*) and barley powdery mildew (*Mla*) are located in syntenic regions on short arm of chromosome. Genome.

[B29] Wehling P, Linz A, Hackauf B, Roux SR, Ruge B, Klocke B (2003). Leaf-rust resistance in rye (*Secale cereale *L.). 1. Genetic analysis and mapping of resistance genes *Pr1 *and *Pr2*. Theor Appl Genet.

[B30] Shi ZX, Chen XM, Line RF, Leung H, Wellings CR (2001). Development of resistance gene analog polymorphism markers for the *Yr9 *gene resistance to wheat stripe rust. Genome.

[B31] Wood EA, Sebesta EE, Starks KJ (1974). Resistance of 'Gaucho' triticale to Schizaphis graminum. Environ Entomol.

[B32] Bennett MD, Gustafson JP, Smith JB (1977). Variation on nuclear DNA in the genus *Secale*. Chromosoma.

[B33] Gaffney T, Friedrich L, Vernooij B, Negrotto D, Nye G, Uknes S, Ward E, Kessmann H, Rayls J (1993). Requirement of salicylic acid for the induction of systemic acquired resistance. Science.

[B34] Draper J (1997). Sugar import and metabolism during seed development. Trends Plant Sci.

[B35] Shirasu K, Nakajima H, Rajasekhar VK, Dixon RA, Lamb C (1997). Salicylic Acid Potentiates an Agonist-Dependent Gain Control That Amplifies Pathogen Signals in the Activation of Defense Mechanisms. Plant Cell.

[B36] Lecerf F, Foggia L, Mulsant P, Bonnet A, Hatey F (2001). A novel method to isolate the common fraction of two DNA samples: hybrid specific amplification (HSA). Nucleic Acids Res.

[B37] Diatchenko L, Lau YF, Campbell AP, Chenchik A, Moqadam F, Huang B, Lukyanov S, Lukyanov K, Gurskaya N, Sverdlov ED, Siebert PD (1996). Suppression subtractive hybridization: A method for generating differentially regulated or tissue-specific cDNA probes and libraries. Proc Natl Acad Sci USA.

[B38] Rogowsky PM, Langridge P, Shepherd KW (1992). Polymerase chain reaction based mapping of rye involving repeated DNA sequences. Genome.

[B39] Clarke BC, Mukai Y, Appels R (1996). The Sec-1 locus on the short arm of chromosome 1R of rye (*Secale cereale *L.). Chromosoma.

[B40] Juttner J, Olde D, Langridge P, Baumann U (2000). Cloning and expression of a distinct subclass of plant thioredoxins. Eur J Biochem.

[B41] Ko JM, Do GS, Suh DY, Seo BB, Shin DC, Moon HP (2002). Identification and chromosomal organization of two rye genome-specific RAPD products useful as introgression markers in wheat. Genome.

[B42] Gu YQ, Coleman-Derr D, Kong X, Anderson OD (2004). Rapid Genome Evolution Revealed by Comparative Sequence Analysis of Orthologous Regions from Four *Triticeae *Genomes. Plant Physiol.

[B43] Kong XY, Gu YQ, You FM, Dubcovsky J, Anderson OD (2004). Dynamics of the evolution of orthologous and paralogous portions of a complex locus region in two genomes of allopolyploid wheat. Plant Mol Biol.

[B44] Wissenbach M (1994). New members of the barley *Kas *gene family encoding beta-ketoacyl-acyl carrier protein synthases. Plant Physiol.

[B45] Rousley S, Linx KK (1998). Large scale sequencing of plant genome. Curr Opin Plant Biol.

[B46] Sasaki T (1998). The rice genome project in Japan. Proc Natl Acad Sci USA.

[B47] Peñuela S, Danesh D, Young ND (2002). Targeted isolation, sequence analysis, and physical mapping of nonTIR NBS-LRR genes in soybean. Theor Appl Genet.

[B48] Spielmeyer W, Huang L, Bariana H, Laroche A, Gill BS, Lagudah ES (2000). NBS-LRR sequence is associated with leaf and stripe rust resistance on the end of homoeologous chromosome group 1S of wheat. Theor Appl Genet.

[B49] Yokoi H, Hadano S, Kogi M, Kang XL, Wakasa K, Joh-Elkeda (1994). Isolation of expressed sequences encoded by the human Xq terminal portion using microclone probes generated by laser microdissection. Genomics.

[B50] Kao FT, Yu J, Tong S, Qi J, Patanjali SR, Weissman SM, Patterson D (1994). Isolation and refined regional mapping of expressed sequences from human chromosome 21. Genomics.

[B51] Yu J (1997). Gene identification and DNA sequence analysis in the GC-poor 20 megabase region of human chromosome 21. Proc Natl Acad Sci USA.

[B52] Wei J, Hodes ME, Wang Y, Feng Y, Ghetti B, Dlouhy SR (1996). Direct cDNA selection with DNA microdissected from mouse chromosome 16: isolation of novel clones and construction of a partial transcription map of the C3–C4 region. Genome Res.

[B53] Yokoyama Y, Ohsugi K, Kozaki T, Sakuragawa N (1997). Microdissection-mediated selection of chromosome region-specific cDNAs. Cytogenet Cell Genet.

[B54] Till BJ, Burtner C, Comai L, Henikoff S (2004). Mismatch cleavage by single-strand specific nucleases. Nucleic Acids Res.

[B55] Hu ZM, Wang H, Chen YH, Shi R, Chen ZH (2003). Studies on contamination of cytoplasm DNA and its control in plant chromosome microdissection. Acta Bot Sin.

[B56] Malamy J, Carr JP, Klessig DF, Raskin I (1990). Salicylic acid: a likely endogenous signal in the resistance response of tobacco to viral infection. Science.

[B57] Yalpani N, León J, Lawton MA, Raskin I (1993). Pathway of salicylic acid biosynthesis in healthy and virus-inoculated tobacco. Plant Physiol.

[B58] Hooft van Huijsduijnen RAM, Alblas SW, de Rijk RH, Bol JF (1986). Induction by salicylic acid of pathogenesis-related proteins and resistance to alfalfa mosaic virus infection in various plant species. J Gen Virol.

[B59] Métraux JP, Signer H, Ryals J, Ward E, Wyss-Benz M, Gaudin J, Raschdorf K, Schmid E, Blum W, Inverardi B (1990). Increase in salicylic acid at the onset of systemic acquired resistance in cucumber. Science.

[B60] Janda T, Szalai G, Tari I, Páldi E (1999). Hydroponic treatment with salicylic acid decreases the effects of chilling injury in maize (*Zea mays *L.) plants. Planta.

[B61] Tan XP, Meyers BC, Kozik A, West ML, Morgante M, Clair DA, Bent AF, Michelmore RW (2007). Global expression analysis of nucleotide binding site-leucine rich repeat-encoding and related genes in Arabidopsis. BMC Plant Biol.

[B62] Zhang K, Martiny AC, Reppas NB, Barry KW, Malek J, Chisholm SW, Church GM (2006). Sequencing genomes from single cells by polymerase cloning. Nat Biotechnol.

[B63] Murray MG, Thompson WF (1980). Rapid isolation of high molecular weight plant DNA. Nucleic Acids Res.

[B64] Nakata N, Yasumuro Y, Sasaki M (1977). An acetocarmine-Giemsa staining of rye chromosomes. Japan J Genetics.

[B65] Telenius H, Carter NP, Bebb CE, Nordenskjöld M, Ponder BAJ, Tunnacliffe A (1992). Degenerated Oligonucleotide-Primed PCR: general amplification of target DNA by a single degenerate primer. Genomics.

[B66] Saghai-Maroof MA, Solman KM, Jorgensen RA, Allard RW (1984). Ribosomal DNA spacer-length polymorphisms in barley: Mendelian inheritance, chromosomal location, and population dynamics. Proc Natl Acad Sci USA.

[B67] Chalaya T, Gogvadze E, Buzdin A, Kovalskaya E, Sverdlov ED (2004). Improving specificity of DNA hybridization-based met hods. Nucleic Acids Res.

[B68] Sambrook J, Russell DW (2001). Molecular Cloning: A Laboratory Manual.

[B69] GenBank. http://www.ncbi.nlm.nih.gov/.

