# Supplementary material for: Rapid EST isolation from chromosome 1R of rye
Source: BMC Plant Biol. 2008 Mar 18;8:28. doi: 10.1186/1471-2229-8-28 (PMC2322994; doi:10.1186/1471-2229-8-28)
Supplement: Additional file 1 — Sequence analysis of ESTs on 1R chromosome of rye plant without SA induction. The data provided the information of sequenced and analyzed ESTs on 1R chromosome of rye plant without SA induction. [file 1471-2229-8-28-S1.doc]

### Supplementary Table1. Sequence analysis of ESTs on 1R chromosome of rye plant without SA induction.

| Clone No. | dbEST_Id | GenBank_Accn | length (bp) | Definition | Score | E value | Identities | Sequence  property |
| --- | --- | --- | --- | --- | --- | --- | --- | --- |
| N1 | 43776779 | EH405881 | 213 | EST from *Secale cereale* (cold stressed) | 373 | 2.00E-100 | 212/221 95% |  |
| N2 | 43782940 | EH412042 | 157 | EST from *Triticum aestivum* | 107 | 1.00E-20 | 100/114 87% | polyA |
| N3 | 43782942 | EH412044 | 78 | EST *Secale cereale* (cold stressed) | 81.8 | 3.00E-13 | 51/57 89% | polyA |
| N4 | 43782953 | EH412055 | 164 | EST from *Triticum aestivum* (Infected with septoria tritici strain A 24 hours) | 264 | 9.00E-68 | 146/149 97% | polyA |
| N5 | 43782943 | EH412045 | 134 | EST from *Hordeum vulgare* | 125 | 4.00E-26 | 104/116 89% | polyA |
| N6 | 43782954 | EH412056 | 152 | *Hordeum vulgare* photosysteme I antenna protein | 301 | 4.00E-79 | 152/152 100% |  |
| N7 | 43802033 | EH431135 | 140 | EST from *Triticum aestivum* | 170 | 9.00E-40 | 116/118 98% | polyA |
| N8 | 43782944 | EH412046 | 291 | *Secale cereale* thioredoxin-like protein | 490 | 2.00E-135 | 271/279 97% | polyA |
| N9 | 43782947 | EH412049 | 256 | EST from *Hordeum vulgare* | 450 | 1.00E-123 | 245/251 97% |  |
| N10 | 43782951 | EH412053 | 155 | EST from *Triticum aestivum* | 163 | 2.00E-37 | 110/118 93% | polyA |
| N11 | 43782948 | EH412050 | 112 | EST *Secale cereale* (cold stressed) | 99.6 | 2.00E-18 | 56/58 96% | polyA |
| N12 | 43782946 | EH412048 | 121 | EST from *Triticum aestivum* (*Fusarium graminearum* inoculated) | 202 | 2.00E-49 | 105/106 99% | polyA |
| N13 | 43782949 | EH412051 | 109 | EST from *Triticum aestivum* (low temperature) | 147 | 9.00E-33 | 89/94 94% | polyA |
| N14 | 43782950 | EH412052 | 105 | Unknown |  |  |  | polyA |
| N15 | 43782955 | EH412057 | 120 | *Hordeum vulgare* partial mRNA for chlorophyll a/b-binding protein. | 188 | 3.00E-45 | 102/103 99% | polyA |
| N16 | 43782956 | EH412058 | 145 | EST from *Triticum aestivum* | 186 | 2.00E-44 | 121/130 93% | polyA |
| N17 | 43782957 | EH412059 | 152 | *Triticum turgidum* A genome HMW glutenin A gene locus, sequence (1A located) | 54 | 2.00E-04 | 51/59 86% |  |
| N18 | 43782958 | EH412060 | 180 | EST from *Triticum turgidum* | 75.8 | 5.00E-11 | 86/102 84% |  |
| N19 | 43782959 | EH412061 | 147 | EST from *Triticum aestivum* (Wheat *Fusarium graminearum* infected) | 52 | 6.00E-04 | 29/30 96% |  |
| N20 | 43782960 | EH412062 | 120 | Unknown |  |  |  |  |
| N21 | 43782961 | EH412063 | 195 | EST from *Secale cereale* (Located chromosome 1R) | 101 | 1.00E-18 | 66/71 92% | polyA |
| N22 | 43782962 | EH412064 | 186 | EST from *Secale cereale* | 56 | 5.00E-05 | 31/32 96% |  |
| N23 | 43782963 | EH412065 | 158 | EST from *Triticum aestivum* (inoculation with *Erysiphe graminis* f. sp tritici) | 81.8 | 7.00E-13 | 47/49 95% |  |
| N24 | 43782964 | EH412066 | 156 | EST from *Triticum aestivum* | 65.9 | 4.00E-08 | 46/49 93% |  |
| N25 | 43782965 | EH412067 | 252 | Unknown |  |  |  |  |
| N26 | 43782966 | EH412068 | 122 | EST from *Secale cereale* | 218 | 4.00E-54 | 110/110 100% | polyA |
| N27 | 43782967 | EH412069 | 200 | EST from *Triticum aestivum* (Fusarium *graminearum* infected) | 347 | 1.00E-92 | 184/187 98% | polyA |
| N28 | 43782968 | EH412070 | 133 | *Hordeum vulgare* photosystem-I PSI-F subunit precursor |  |  |  | polyA |
| N29 | 43782970 | EH412072 | 157 | EST from *Triticum aestivum* | 309 | 2.00E-81 | 156/156 100% |  |
| N30 | 43782971 | EH412073 | 76 | *Triticum aestivum* cold acclimation protein | 139 | 2.00E-30 | 70/70 100% |  |
| N31 | 43782973 | EH412075 | 154 | EST from *Secale cereale* (cold stressed) | 256 | 2.00E-65 | 153/162 94% |  |
| N32 | 43782952 | EH412054 | 122 | EST from *Secale cereale* (Irradiance or Excitation Pressure) | 188 | 3.00E-45 | 95/95 100% | polyA |
| N33 | 43782941 | EH412043 | 224 | EST from *Secale cereale* (Temperature, Irradiance or Excitation Pressure) | 416 | 1.00E-113 | 210/210 100% | polyA |
| N34 | 43782945 | EH412047 | 94 | Unknown |  |  |  |  |
| N35 | 43782969 | EH412071 | 78 | EST from *Secale cereale* | 99.6 | 1.00E-18 | 57/58 98% | polyA |
| N36 | 43782975 | EH412077 | 369 | Unknown |  |  |  |  |
| N37 | 43782972 | EH412074 | 80 | EST from *Triticum turgidum* | 83.8 | 8.00E-14 | 57/62 91% | polyA |
| N38 | 43782977 | EH412079 | 89 | *Secale cereale* OPH20 RAPD marker sequence (located chromosome 1R-7R) | 109 | 1.00E-21 | 63/65 96% |  |
| N39 | 43782978 | EH412080 | 98 | EST from *Secale cereale* | 157 | 8.00E-36 | 82/83 98% | polyA |
| N40 | 43782979 | EH412081 | 78 | *H. vulgare* mRNA for cystein proteinase inhibitor | 97.6 | 5.00E-18 | 60/63 95% | polyA |
| N41 | 43782981 | EH412083 | 130 | Unknown |  |  |  | polyA |
| N42 | 43782976 | EH412078 | 62 | EST from *Triticum aestivum* | 93.7 | 6.00E-17 | 47/47 100% | polyA |
| N43 | 43782982 | EH412084 | 112 | EST from *Triticum aestivum* (low temperature) | 143 | 2.00E-31 | 90/96 93% | polyA |
| N44 | 43782983 | EH412085 | 173 | Unknown |  |  |  |  |
| N45 | 43782984 | EH412086 | 149 | *H. vulgare* (pMaW25) mRNA for beta-ketoacyl-ACP synthase | 204 | 7.00E-50 | 128/134 95% | polyA |
| N46 | 43782985 | EH412087 | 245 | EST from *Aeluropus lagopoides* (drought-treated ) | 165 | 1.00E-37 | 124/135 91% |  |
| N47 | 43782980 | EH412082 | 214 | Unknown |  |  |  |  |
| N48 | 43782988 | EH412090 | 135 | Unknown |  |  |  |  |
| N49 | 43782987 | EH412089 | 411 | EST from *Hordeum vulgare* | 121 | 2.00E-24 | 106/121 87% |  |
| N50 | 43782989 | EH412091 | 303 | Unknown |  |  |  |  |
| N51 | 43782986 | EH412088 | 98 | EST from *Hordeum vulgare* | 61.9 | 4.00E-07 | 31/31 100% |  |
| N52 | 43782974 | EH412076 | 98 | *Triticum aestivum* cultivar Triple Dirk D line VRN-A1 (VRN-A1) gene | 58 | 6.00E-06 | 32/33 96% |  |
| N53 | 43782992 | EH412094 | 80 | EST from Hordeum vulgare |  |  |  |  |
| N54 | 43782991 | EH412093 | 150 | *H. vulgare* (pMaW25) mRNA for beta-ketoacyl-ACP synthase | 212 | 3.00E-52 | 132/138 95% | polyA |
| N55 | 43782993 | EH412095 | 218 | EST from *Triticum aestivum* | 182 | 4.00E-43 | 165/186 88% | polyA |
| N56 | 43782990 | EH412092 | 111 | EST from *Triticum aestivum* (treated with 6-iodo-3-propyl-2-propyloxy-4 (3H)-quinazolinone) | 190 | 7.00E-46 | 96/96 100% | polyA |
| N57 | 43782994 | EH412096 | 114 | Unknown |  |  |  |  |
| N58 | 43782995 | EH412097 | 140 | Unknown |  |  |  |  |
| N59 | 43782996 | EH412098 | 189 | EST from *Triticum aestivum* (cold accumulated) | 196 | 2.00E-47 | 168/187 89% |  |
| N60 | 43782997 | EH412099 | 312 | EST from *Triticum aestivum* (treated with 6-iodo-3-propyl-2-propyloxy-4 (3H)-quinazolinone) | 111 | 1.00E-21 | 119/139 85% |  |
