# Supplementary material for: Rapid EST isolation from chromosome 1R of rye
Source: BMC Plant Biol. 2008 Mar 18;8:28. doi: 10.1186/1471-2229-8-28 (PMC2322994; doi:10.1186/1471-2229-8-28)
Supplement: Additional file 2 — Sequence analysis of ESTs on 1R chromosome of rye plant with SA induction. The data provided the information of sequenced and analyzed ESTs on 1R chromosome of rye plant with SA induction. [file 1471-2229-8-28-S2.doc]

### Supplementary Table 2 -Sequence analysis of ESTs on 1R chromosome of rye plant with SA induction.

| Clone No. | dbEST_Id | GenBank_Accn | length (bp) | Definition | Score | E value | Identities | Sequence  property |
| --- | --- | --- | --- | --- | --- | --- | --- | --- |
| SA1 | 43783012 | EH412114 | 219 | EST from *Triticum turgidum* | 77.8 | 2.00E-11 | 73/82 89% |  |
| SA2 | 43783003 | EH412105 | 208 | *Secale cereale* omega secalin gene (Located chromosome 1Rs) | 98 | 9.00E-47 | 142/154 92% |  |
| SA3 | 43783015 | EH412117 | 167 | Unknown |  |  |  |  |
| SA4 | 43783016 | EH412118 | 183 | Unknown |  |  |  |  |
| SA5 | 43783017 | EH412119 | 186 | Unknown |  |  |  |  |
| SA6 | 43783018 | EH412120 | 189 | Unknown |  |  |  |  |
| SA7 | 43783019 | EH412121 | 219 | *Triticum turgidum* HMW-glutenin locus (Located chromosome 1B) | 141 | 1.00E-30 | 183/219 83% |  |
| SA8 | 43783021 | EH412123 | 168 | Wheat major chlorophyll a/b-binding protein gene | 278 | 6.00E-72 | 155/160 96% |  |
| SA9 | 43783022 | EH412124 | 119 | EST from *Triticum aestivum* | 143 | 2.00E-31 | 93/100 93% | polyA |
| SA10 | 43783023 | EH412125 | 187 | *Triticum aestivum* putative glycine decarboxylase subunit | 307 | 8.00E-81 | 168/171 98% | polyA |
| SA11 | 43783020 | EH412122 | 60 | EST from *Triticum aestivum* | 83.8 | 5.00E-14 | 45/46 97% | polyA |
| SA12 | 43783024 | EH412126 | 148 | EST from *Triticum aestivum* (cold stress) | 163 | 2.00E-37 | 112/122 91% | polyA |
| SA13 | 43783025 | EH412127 | 144 | Unknown |  |  |  | polyA |
| SA14 | 43783026 | EH412128 | 95 | EST from *Triticum aestivum* (drought stress) | 105 | 3.00E-20 | 59/60 96% | polyA |
| SA15 | 43783027 | EH412129 | 66 | EST from *Triticum aestivum* (cold stress) | 85.7 | 2.00E-14 | 49/51 96% | polyA |
| SA16 | 43783028 | EH412130 | 127 | EST from *Secale cereale* (cold stressed) | 161 | 7.00E-37 | 105/113 92% | polyA |
| SA17 | 43783029 | EH412131 | 151 | EST from *Triticum aestivum* (cold stress) | 159 | 4.00E-36 | 118/126 91% | polyA |
| SA18 | 43783002 | EH412104 | 104 | EST from *Secale cereale* (cold stressed) | 157 | 9.00E-36 | 79/79 100% |  |
| SA19 | 43782999 | EH412101 | 109 | EST from *Triticum turgidum* | 153 | 2.00E-34 | 89/92 96% | polyA |
| SA20 | 43783030 | EH412132 | 93 | EST from *Secale cereale* (cold stressed) | 145 | 3.00E-32 | 73/73 100% | polyA |
| SA21 | 43783031 | EH412133 | 145 | EST from *Hordeum vulgare* | 258 | 5.00E-66 | 130/130 100% | polyA |
| SA22 | 43783004 | EH412106 | 62 | EST from *Triticum aestivum* | 87.7 | 4.00E-15 | 44/44 100% | polyA |
| SA23 | 43783000 | EH412102 | 84 | Unknown |  |  |  | polyA |
| SA24 | 43783013 | EH412115 | 80 | EST from *Triticum aestivum* (inoculation with leaf rust pathogen *Puccinia triticina*) | 113 | 9.00E-23 | 60/61 98% | polyA |
| SA25 | 43783005 | EH412107 | 74 | Unknown |  |  |  | polyA |
| SA26 | 43783007 | EH412109 | 111 | EST from *Triticum aestivum* | 131 | 6.00E-28 | 91/95 92% | polyA |
| SA27 | 43783006 | EH412108 | 111 | EST from *Hordeum vulgare* | 131 | 6.00E-28 | 81/86 94% | polyA |
| SA28 | 43783008 | EH412110 | 146 | Unknown |  |  |  | polyA |
| SA29 | 43783009 | EH412111 | 127 | *Triticum aestivum* cold acclimation protein | 163 | 2.00E-37 | 88/90 97% | polyA |
| SA30 | 43783010 | EH412112 | 152 | *H. vulgare* (pMaW25) mRNA for beta-ketoacyl-ACP synthase | 163 | 2.00E-37 | 101/105 96% | polyA |
| SA31 | 43783011 | EH412113 | 163 | Unknown |  |  |  |  |
| SA32 | 43782998 | EH412100 | 199 | EST from *Secale cereale* (aluminum-stressed) | 309 | 2.00E-81 | 156/156 100% | polyA |
| SA33 | 43783014 | EH412116 | 200 | EST from *Triticum aestivum* | 117 | 2.00E-23 | 115/133 86% | polyA |
| SA34 | 43783001 | EH412103 | 133 | Unknown |  |  |  | polyA |
